# Stop the pain: study protocol for a randomized-controlled trial

**DOI:** 10.1186/1745-6215-15-357

**Published:** 2014-09-11

**Authors:** Petra Warschburger, Claudia Calvano, Sebastian Becker, Michael Friedt, Christian Hudert, Carsten Posovszky, Maike Schier, Karl Wegscheider

**Affiliations:** Department Psychology, Counseling Psychology, University of Potsdam, Karl-Liebknecht-Straße 24-25, Potsdam, 14476 Germany; Paediatric Gastroenterology, Princess Margaret Children’s Hospital Darmstadt, Dieburger Str. 31, Darmstadt, 64287 Germany; Department of General Paediatrics, Neonatology and Paediatric Cardiology, Division of Paediatric Gastroenterology, University Children’s Hospital, Moorenstr. 5, Duesseldorf, D-40225 Germany; Department of Gastroenterology, Charité University Medicine, Virchow Campus, Augustenburger Platz 1, Berlin, 13353 Germany; Department of Paediatrics and Adolescent Medicine, University Medical, Centre Ulm, Eythstr. 24, 89075 Ulm, Germany; Department of Gastroenterology, Catholic Children’s Hospital Wilhelmstift, Hamburg, Germany; Department of Medical Biometry and Epidemiology, University Medical, Centre Hamburg-Eppendorf, Martinistr. 52, Hamburg, 20246 Germany

**Keywords:** FAP, Randomized controlled trial, Cognitive behavioral intervention, Children, Pain

## Abstract

**Background:**

Functional abdominal pain (FAP) is not only a highly prevalent disease but also poses a considerable burden on children and their families. Untreated, FAP is highly persistent until adulthood, also leading to an increased risk of psychiatric disorders. Intervention studies underscore the efficacy of cognitive behavioral treatment approaches but are limited in terms of sample size, long-term follow-up data, controls and inclusion of psychosocial outcome data.

**Methods/Design:**

In a multicenter randomized controlled trial, 112 children aged 7 to 12 years who fulfill the Rome III criteria for FAP will be allocated to an established cognitive behavioral training program for children with FAP (n = 56) or to an active control group (focusing on age-appropriate information delivery; n = 56). Randomization occurs centrally, blockwise and is stratified by center. This study is performed in five pediatric gastroenterology outpatient departments. Observer-blind assessments of outcome variables take place four times: pre-, post-, 3- and 12-months post-treatment. Primary outcome is the course of pain intensity and frequency. Secondary endpoints are health-related quality of life, pain-related coping and cognitions, as well as selfefficacy.

**Discussion:**

This confirmatory randomized controlled clinical trial evaluates the efficacy of a cognitive behavioral intervention for children with FAP. By applying an active control group, time and attention processes can be controlled, and long-term follow-up data over the course of one year can be explored.

**Trial registration:**

DRKS00005038 (date: 25 July 2013); NCT02030392 (date: 7 January 2014)

**Electronic supplementary material:**

The online version of this article (doi:10.1186/1745-6215-15-357) contains supplementary material, which is available to authorized users.

## Background

Functional abdominal pain (FAP) is a chronic pain disorder of the gastrointestinal tract without underlying pathological condition [1]. According to the Rome III criteria, functional abdominal pain is specified as functional dyspepsia, irritable bowel syndrome, functional abdominal pain, and functional abdominal pain syndrome [2]. FAP is characterized by recurrent or persistent pain in the abdomen for more than 2 months, with episodes occurring at least once a week [2]. FAP represents, besides headache, the most common pain syndrome in childhood [3], with prevalence rates ranging from 8.3% [3] up to 45% [2]. FAP often leads to functional disability [4], poor school attendance [4] and a number of unnecessary, cost-intensive visits in medical care settings [5]. Children with recurrent episodes of abdominal pain may have a worse quality of life [6] and self-esteem [4] and experience greater anxiety and depression [7, 8]. Untreated, FAP is highly persistent until adulthood, also leading to increased risk of psychiatric disorders (see [9]).

Psychosocial factors in symptom maintenance include inadequate coping styles [10], poor family functioning and overprotective parental behaviors [11]. Catastrophizing plays an especially important role in mediating the relationship between pain and quality of life [6] and moderating the effectiveness of distraction [12].

Systematic reviews and meta-analyses underscore the efficacy of psychological interventions, especially cognitive-behavioral therapy (CBT), for managing pain in childhood and adolescence [13–18]. But only a few treatment studies for pediatric chronic pain consider abdominal pain (3 out of 25; see [16]) and even fewer investigate the emotional functioning of the children [16, 18]. A recent meta-analysis by Sprenger *et al*. [18], including ten studies for the psychological treatment of FAP, showed that interventions obtain a medium-sized effect on pain reduction. These interventions mainly comprised CBT, child-centered or family-based, and relaxation techniques. With respect to functional and emotional outcomes, CBT involving the children alone [19] or parents and children [11, 20–22] proved to be effective in terms of an increase in emotional functioning or in quality of life. Interpretation of studies is limited due to differing definitions of the condition, inadequate control group designs and a lack of assessment of long-term effects [16]. With respect to longer term follow-up and the effectiveness of CBT compared to an active control group, available evidence is limited. Since preparation of this trial, several RCT studies with 12-month follow-up data have been published [23, 24] but with inconsistent results. While Levy *et al*. [11, 23] report a greater decrease in pain symptoms and parental solicitous behavior, as well as greater increase in child’s coping skills in the intervention group compared to the active control group, results of the study by van der Veek *et al*. [24] could not confirm these differences. Comparing a cognitive-behavioral, child-centered intervention and intensive medical care, van der Veek *et al*. [24] found no group differences in pain reduction at post-treatment and at the 12-month follow-up. All secondary outcomes, for example, quality of life, improved equally in both groups.

The current trial builds on the insights gained in two previous studies. For example, in an uncontrolled pilot study with 11 children, we were able to show that children attending a cognitive-behavioral program [20] not only experienced a reduction in pain frequency and intensity, but also an increase in health-related quality of life. A small RCT-study (n = 29; including a waiting list control group) was able to confirm these results with effect sizes ranging from medium to high for a 3-month follow-up period [21, 22].

Taken together, these data suggest that cognitive-behavioral interventions are effective for pain reduction; however little is known about the differential effects on emotional well-being and individual differences in treatment response.

This multicenter trial aims to compare the cognitive-behavioral group intervention with an active control group (age-appropriate educational content), both provided by trained therapists, with respect to a reduction of pain symptoms and an increase in children’s quality of life. The effects are analyzed 3 and 12 months after the end of treatment.

## Methods/Design

### Study design

**Figure 1:**
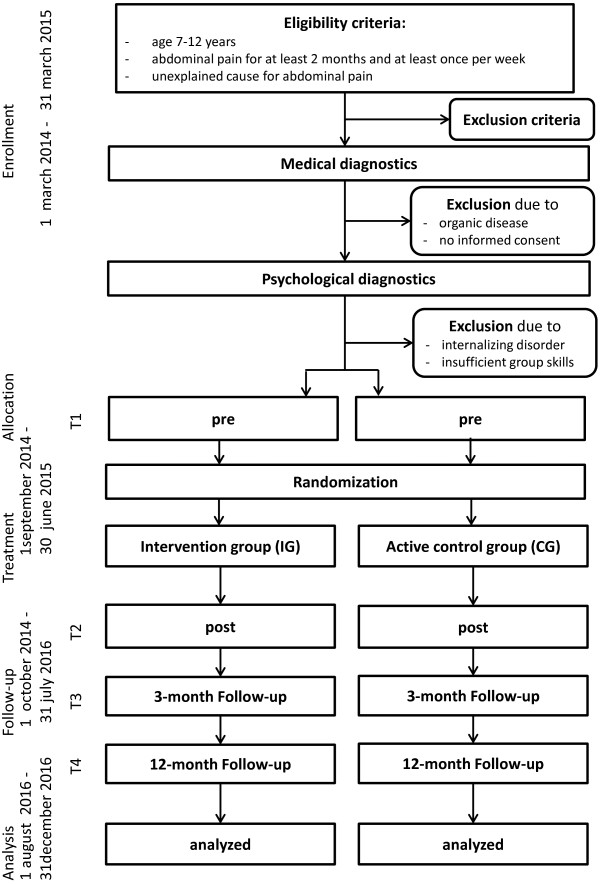
**Trial flow chart.**

Stop-FAP is a multicenter, prospective, parallel group, randomized-controlled superiority trial for the evaluation of the efficacy of a cognitive-behavioral group intervention compared to an active control group. Study process from enrollment to follow-up is depicted in Figure 1 (Trial Flow Chart).

#### Study setting

This study is conducted in five study centers, all of which are outpatient clinics for pediatric gastroenterology (Berlin, Darmstadt, Düsseldorf, Hamburg, Ulm). Participating study centers and investigators are fully listed in the German Clinical Trials Register (DRKS00005038) and in the registry of the U.S. National Institutes of Health clinicaltrials.gov (NCT02030392).

### Recruitment

After initiation of the study centers, the 12-month recruitment phase of this ongoing trial started in March 2014. Patients will be recruited consecutively during regular consulting hours. Supplementary recruitment strategies involve active recruitment by information flyers and posters or by informing former patients about the study.

### Participants

Study inclusion follows a stepwise procedure. In the first step we defined eligibility criteria for medical screening as listed below:

#### Medical screening

Inclusion criteria include the following:

1. age 7 to 12 years,
2. Rome III criteria for FAP (that is, abdominal pain at least once per week for at least two months), and
3. unexplained cause for abdominal pain.

Exclusion criteria include the following:

1. insufficient German language skills,
2. mental retardation,
3. physician-ordered medication or therapy (psychological, pharmacological) of gastrointestinal complaints at the screening visit,
4. participation in a clinical trial that may have effects on gastrointestinal symptoms in the preceding 4 weeks,
5. participation in a training program for gastrointestinal complaints in the preceding 6 months, or
6. presentation of a sibling aged 7 to 12 years suffering from abdominal pain.

The standardized procedure of gastroenterological diagnostics is explained in more detail in the section ‘procedures’ below. In the second step, we defined inclusion criteria for study participation as follows:

1. functional abdominal pain (FAP) presenting as functional dyspepsia, irritable bowel syndrome, functional abdominal pain or functional abdominal pain syndrome (Rome-III Criteria H2a, H2b, H2d, H2d1), and
2. informed written consent of parent(s) and child.

Exclusion criteria for study participation are as follows:

1. severe internalizing psychiatric disorders with primary treatment indication, or
2. very limited group skills that are operationalized as severe externalizing disorders.

Sites (n = 5) were selected based on the availability of a pediatric gastroenterological consultation hours and staff experienced in diagnosis and treatment for FAP (for example, certified by the German Society for Pediatric Gastroenterology and Nutrition, GPGE). Eligibility criteria for the selection of trainers (n = 10) were expertise and occupational background for pediatric psychology, chronic pain, child psychotherapy or treatment of children with chronic diseases. All trainers received an intensive training in conducting both interventional and control training (IG and CG) during a mandatory 3-day train-the-trainer seminar.

### Procedures: identification of trial participants

**Figure 2:**
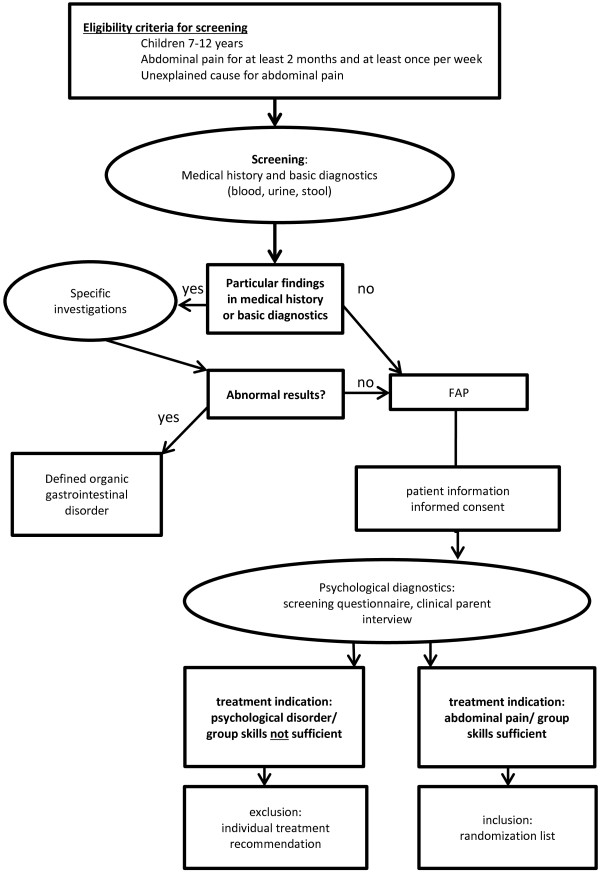
**Procedures: identification of trial participants.**

Prior to study inclusion, children will undergo standardized medical and psychological examination to exclude organic gastrointestinal disorders in the first step and psychiatric disorders with primary treatment indication in the second step (see Figure 2). Information about pain symptoms and associated factors (somatoform symptoms and family history of pain) is collected during the screening process by parent questionnaire. We aim to screen n = 450 children.

#### Medical screening

Medical screening for exclusion of organic gastrointestinal disorders is standardized across study centers and follows current guidelines [1, 25, 26]. Following a stepwise approach, the physician will clarify red flags by a standardized medical history (related symptoms, fever, blood in the urine, waking up during night, bowel habits, nausea *etcetera*), thorough examination and laboratory diagnostics (that is, blood, urine status, and stool), all of which are obligatory and necessary steps to rule out organic causes of pain such as inflammatory bowel disease [1, 25, 26]. In case of any abnormality, further diagnostics will be conducted accordingly (for example, elimination diet in case of signs of food intolerance). According to the Rome III criteria, in case of no pathological result, a diagnosis of FAP can be established. At this point, information about the trial will be given to the child and parents, and written consent will be obtained by the site investigator.

#### Psychological screening

In the next step, the child will be referred for psychological screening, to rule out primary severe psychological problems associated with abdominal pain. The psychological diagnostics follow a two-step procedure, with a standardized and validated screening questionnaire (SDQ; [27–29]) for all parents of eligible children in the first step. In case of subclinical or clinical scores on the scales for emotional problems, hyperactivity or conduct problems, a structured clinical interview (Kinder-DIPS; [30]) with the parent will be held to verify the reported abnormalities (see [31]), to determine psychological strain, group skills and treatment indication.

Since up to 45% of the FAP children suffer from psychological problems - mainly in the subclinical range [7, 8] - we do include children with subclinical psychological disorders to ensure that we are able to collect a representative study sample. Children with clinically relevant psychiatric symptoms will only be included when FAP is declared to be associated with the highest psychological strain leading to primary treatment indication.

### Intervention and **c**ontrols

Intervention group (IG) and control group (CG) are comparable with regard to group size (3 to 8 children), number and duration of sessions for children (n = 6, 90 min each) and parents (n = 2, 50 min each). Training sessions for children are once a week. In both groups, the intervention period per patient lasts 6 weeks in the study centers, with 2 weeks pre- and postintervention being added for the pain diary. All study centers conduct both treatment approaches.

#### Standardization

Both experimental and control intervention are manualized programs for maximizing standardization and improving protocol adherence.

#### Intervention group - stop the pain with happy pingu

Children and their parents randomized to the IG will participate in the cognitive behavioral self-management program ‘Stop the Pain with Happy Pingu’ [32]. The standardized and manualized intervention addresses the following issues: psychoeducation on FAP, change of negative pain-related thoughts and attention bias, relaxation techniques, coping strategies and self-esteem. Each session includes individual tailoring of content, for example, triggers of pain. A central element of the program is the pain diary that children are asked to keep during the whole intervention in order to increase their self-management skills. Each session is accompanied by regular homework (for example, relaxation) that allows implementing and practicing of the newly learned strategies at home. The child’s experiences can be tackled in the following session.

During the parent group sessions, the parents are primarily informed about the major disease characteristics, the etiology and maintenance of FAP, red flags and nutrition. Main focus of the parental sessions is to coach the parents on how to support their child in his pain management efforts.

#### Control group - educational program

The CG comprises a child-centered educational program [33, 34]. The sessions will include information mainly about the gastrointestinal tract, nutrition and bodily activity. Adverse side effects of this program are not reported [20–22]. In order to measure the course of pain severity continuously, the CG will fill in the pain diary as weekly homework. For ethical reasons, we will inform the parents about the so-called red flags that indicate the need for contacting a medical doctor [1, 25]. All parents will be reassured that there is no serious organic disease present and that the pain of their child is real. There will be no recommendations by the therapist for any food or diet and neither parent nor child will receive a specific training to cope with pain.

#### Treatment integrity and protocol adherence

The quality of the intervention is assured by the standardized manual and a mandatory 3-day train-the-trainer seminar for all the persons conducting the intervention, followed by ongoing supervision via regular telephone calls (once a month) and conferences (every 3 months) and monitoring on the spot.

By checking trainer protocols of sessions, adherence to intervention protocols will be improved by giving feedback to the trainers. In addition, all sessions in both the IG and CG will be recorded by audiotapes. Analysis with respect to protocol violations will be conducted by a trained person blind to treatment allocation.

### Hypotheses

Our primary hypothesis postulates that children in the IG will experience a more pronounced and sustained reduction of the frequency and intensity of abdominal pain than children in the CG.

Secondary hypotheses postulate that after treatment, children in the intervention group - compared to an active control group - will experience (i) higher improvement in health- and pain-related quality of life, (ii) a higher increase in the use of adequate pain-related coping and cognitions, and (iii) a more pronounced improvement in self-efficacy.

Additionally, we assume that children and parents with more pronounced psychosocial strain gain less from treatment than those experiencing low strain.

### Outcome variables

#### Primary outcome measure

The primary outcome is the course of pain frequency and intensity of FAP (composite score) from pretreatment (T1) to post-treatment (T2), 3-month (T3) and 12-month follow-up (T4). Pain symptoms will be assessed via self-report using the pain diary, which has already successfully been implemented in prior trials [20–22]. On 14 consecutive days, children should report intensity of pain on a 10-point visual analog scale. Pain duration and frequency are derived from reports on time spans of when the pain arose.

#### Secondary outcome measure

Psychosocial well-being (health-related quality of life) and pain coping, cognitions and self-efficacy are of clinical relevance and represent the secondary outcomes. All secondary outcomes are assessed by validated questionnaires as self or proxy reports.

#### Heath-related quality of life

Health-related quality of life (HRQoL) is assessed by the German version of the Pediatric Quality of Life Inventory. (PedsQL; [35]). This 23-item questionnaire covers four dimensions of HRQoL (physical, emotional, school, and social). This measure showed sufficient to good internal consistency in previous studies (α = .68 to α = .80; [20, 21]). In addition, we assess the child’s pain-related quality of life by the illness module of KINDL^R^ [36], which covers six items. Children are to rate the degree of difficulties for each item on a five-point scale (never to almost always). HRQoL will be assessed in both the child and parent report.

#### Pain - related coping and cognitions

Pain-related coping will be assessed by the German version of the Pediatric Pain Coping Inventory (PPCI-revised; [37, 38]). The questionnaire includes three subscales of behavior-related coping strategies when the child is in pain: passive pain coping (for example, ‘I go to bed’ (10 items, α = .68)), seek for social support (for example, ‘I have my mother, father or a friend sit with me’ (8 items, α = .80)) and positive self-instruction (for example, ‘I tell myself to be brave’ (7 items, α = .63)).

#### Self-efficacy

For assessing child’s pain-related self-efficacy, we use the self-efficacy scale by Bursch [39]. Children are asked to rate how sure they are to pursue everyday activities when they are in pain (for example, ‘make it through a school day’ or ‘be with your friends’) on a five-point scale (very sure to very unsure). This seven-item measure showed good internal consistency in the validation study (α = .80; [39]).

#### Predictors

In addition to the confirmatory aspects of this trial, further child- and parent-related variables will be assessed for analyzing predictive or moderating influences on treatment effects. Child and parental strain will be analyzed with respect to their impact on treatment outcome.

Further, the amount of healthcare utilization and school attendance will be assessed at baseline and during follow-up.

#### Points of measurement

Applying an observer-blind design, data are collected by questionnaires for children and parents at four points of measurement. Pre-intervention (T1) and postintervention (T2) will take place at the study centers. As the primary outcome is assessed by a pain diary over 14 days, T1 and T2 are located 14 to 21 days before and after the intervention phase. Follow-up assessment will take place 3 (T3) and 12 (T4) months after intervention, also including pain diaries and questionnaires for child and parents. Follow-up data will be collected via mail by the University of Potsdam.

The issue of concomitant care and use of other medical or psychological services is included in patient information prior to study inclusion. The families are not to utilize other therapeutic approaches for FAP such as pain medication or other treatments. Due to ethical reasons, medical or psychological consultations are not prohibited during the follow-up time span. Data on the consultation rate are included in the follow-up survey.

### Sample size - power analysis

The calculations rely on the high effect size of our primary outcome (d = 1.09 for the composite pain score; see [21, 22]) and correlation among repeated measures 0.8 (see [20–22]). Since our recent single-site study included a 3-month follow-up and a waiting list control group (instead of an active control group [21, 22]), power analysis for the primary outcome is based conservatively on a medium effect size (f = 0.25; d = 0.6). Independent variables are intervention (2), gender (2), age (2) and site of intervention (5), forming a total of 2^3^*5 = 40 groups.

In the study centers, n = 450 children will be assessed for eligibility and a total number of n = 112 children will be allocated to the trial (n = 56 in IG, n = 56 in CG). We assume a drop-out rate of 20% [cf. 18], resulting n = 112 children to be analyzed in the intention-to-treat analysis (ITT) and n = 90 children for the per protocol analysis (PPA).

#### Drop-out rate

In our pilot study we did not observe drop-out of patients. In 9 out of 10 intervention studies, drop-out rates are below 25% [18]. We assume a loss of 20% due to drop-outs during the follow-up time span (cf. [40]). To prevent selective drop-out and incomplete outcome data, all participants will be reimbursed for completely filling in the questionnaires and the pain diary in the follow-up period.

### Randomization

Allocation sequences are generated centrally at the Institute of Epidemiology at University Clinic of Hamburg-Eppendorf (KW). It will be mutually randomized in IG and CG with a 1:1 ratio. Each patient’s position on allocation sequence is defined by computer-generated random-numbers and the date of informed consent (chronologically sorted).

As groups are assigned parallel and the interventions are to be carried out mutually, blocking is dependent on the group sizes. Block sizes range between 6 and 16 patients. The total number of each block is dependent on the total number of groups to be trained. As in this trial, a maximum of 25 groups are to be trained; the number of blocks of 6 and 8 patients is restricted to five each.

The randomization procedure, in order to prevent selection bias, occurs centrally at the University of Potsdam by a person not involved in the intervention process and data analysis. We realize a stratified (according to center) block randomization. Randomization and allocation to interventions occur after assessment of pre-data and before the interventions start. Results of randomization will be provided directly only to the trainers via email (allocation concealment). Each center has to realize four different groups; at the first block of interventions, the trainer may choose the trial arm (IG versus CG); in the second block, the trainers have to change the trial arm.

The effectiveness of the randomization is evaluated post-hoc by descriptive statistical comparisons between IG and CG in known and presumed baseline predictors of treatment success (for example, pain history of the parents and socio-economic status) and relevant sociodemographic data (age and sex).

### Blinding

The medical and psychological assessments are blinded as these precede randomization. After assignment to interventions, the outcome assessors (study nurse and site investigators) and principle investigator will be blinded. While patients and parents will be blinded, blinding of trainers in the treatment is not possible. However, trainers are not involved in the assessment process.

Group allocation will be coded by different letters; only the trainers and the administrator of randomization results will be informed about the corresponding intervention.

The primary outcome will be reported by the children themselves, who are not aware of their respective treatment allocation. Assessment of data is blinded on all points of measurement. Follow-up data will be assessed by telephone interviews (blinded interviewer) with the younger children and questionnaires for older children and the parents. Information material and questionnaires in the follow-up phase will be sent out directly to the participating families. Data collection and data analysis are independent. Data entry is operated by computer scanning and is therefore blinded.

Early unblinding is not intended. Regular unblinding occurs after complete data collection, that is, after the last data of T4 is collected and entered into data set. Process data of the intervention-specific pain diaries will be added to the data set after all other data are entered and therefore constitutes the first step of unblinding.

### Stopping rules

For the individual patient, it is stated that children can discontinue treatment in case of observed unusual, continued and increased, severe bodily complaints or in case of red flags. Then, the study physician will be consulted, and diagnostics and treatment are advised. The whole intervention will be stopped in case of occurrence of severe adverse effects in association with the intervention in more than 5% of the children. A study center will be closed when it fails to meet or adhere to the study protocol in more than three cases in a row, or if for two consecutive time spans, fewer than three children could be included in the study over the course of 3 months.

### Ethical approval

This trial was approved by the Ethics Committee of the University of Potsdam and the responsible ethics committees/state chambers of physicians of the five study centers. A list of ethical bodies can be found in Additional file 1.

All study staff commit themselves to the Declaration of Helsinki (Version 2013), as well as to all pertinent national laws and the ICH guidelines for GCP issued in June 1996 and CPMP/ICH/ 135/95 from July, 2002. Important protocol modifications will be reported to all relevant parties.

### Data safety

Staff working in this trial will be bound by the duty of confidentiality. The respondents’ privacy and anonymity is safe-guarded as personal patient data will be stored in a restricted room separately from the questionnaires. The latter are coded in an anonymous way before their transfer to the central study office where data entry and analysis will take place. All personalized data (addresses) are destroyed after the termination of follow-up. The participating treatment facilities will only receive aggregated statistical data as feedback. All potential study participants receive adequate written information about the aims and implications of the study before consent is given. Informed consent will be obtained from children and their parents by the site investigators.

### Data analytic plan

Missing values will be replaced by unbiased substitution (EM-algorithm). Data will be analyzed by intention to treat (ITT) supplemented by a per protocol analysis (PPA) to test for effects of treatment adherence.

### Primary analysis of the primary endpoints

An analysis of covariance will be performed in the ITT population, comparing the log area under the pain intensity curve from intervention to 12-month follow-up between the two random groups adjusted for the baseline measurements. Length of pain episodes will be analyzed analogously. Frequencies will be analyzed using a Poisson regression model.

### Confirmatory analysis

In terms of clinical relevance, the composite pain score is the most prominent outcome: a substantial pain reduction is mandatory for treatment efficacy. We therefore apply a hierarchical testing procedure; the number and length of pain episodes will be tested only if there is a significant difference between the treatment groups in the log area under the curve (auc) of pain intensity. These endpoints will be tested to an alpha level of 2.5%; therefore, under this hierarchical design the overall alpha level of the trial will not exceed 5%.

### Analysis of secondary outcomes

Further, we will analyze primary and secondary outcome measures using a mixed model, adjusting for the therapist (random effect) and baseline measures (including age, gender, duration of illness, age at onset and the baseline measure of the outcome of interest).

### Exploratory analyses

We will also explore subscales of the outcome measures to see if there are indicators of treatment effects within these subscales.

In further exploratory analyses, we will include other baseline predictors in the model and explore their potential effect modification; moreover the effect of time-varying predictors will also be investigated. In a sensitivity analysis, we will explore the effect of missing values using various imputing methods (best and worst observation carried forward, last observation carried forward, full-information maximum likelihood and multiple imputation).

### Descriptive analysis

All available data will be analyzed descriptively for each intervention group. Differences in baseline variables will be analyzed by appropriate tests (for example, the Chi squared test, Kruskal-Wallis test, or analyses of variance) in pairwise comparisons between the two treatment groups.

### Monitoring and data management

We concentrate our quality assurance and monitoring on two aspects: standardization of recruitment and data collection, and standardization/fidelity of intervention.

#### Monitoring

Regular monitoring on the spot includes supervision of study nurses, control of protocol adherence, adherence to intervention protocol and data quality assurance.

In addition, for coordinating the trial across the study centers, there will be, in addition to the above mentioned monitoring on the spot, principle investigator (PI) and site investigator conference calls for cross-site reliability, standardization and monitoring by checking compliance to the protocol, inclusion/exclusion criteria and progress of the intervention (individually, every month and for all trial sites and the coordinator, every 3 months). For assuring the quality of treatment, the therapist and evaluator will be in contact via conference calls as well.

Follow-up assessment is monitored by the principle investigator. Audits can be held by the funding organization at any time and the process will be independent from the sponsor and the investigators.

#### Data management

Database management, randomization and the biometrical calculations will be carried out at the Department of Medical Biometry and Epidemiology (KW) and University of Potsdam (PW, CC). The study will be reported according to the CONSORT criteria for non-pharmacological trials [41] and will be supplemented according to the TIDieR guidelines [42]. For all patients, the informed consent documents will be checked. Data entry will be conducted by electronic scanning of questionnaires, which includes double checking of data. Additionally, 20% of the data will be double checked by staff. The primary outcome measures will be verified in 100% of cases. In addition, source data verification of the key data will be performed in a random sample of 40.0% of the patients. Data management has the opportunity to conduct queries regarding data quality and integrity.

#### Assessment of safety

Since we do not expect adverse effects of the psychological intervention, we decided to form a multidisciplinary scientific advisory board (Trial Steering Committee, TSC) to oversee and monitor the whole trial. The members are experts in the field of chronic pain and will supervise the trial to ensure data safety. The TSC will receive recruitment and retention updates on a regular basis. According to the nature of the study, those persons monitor the progress of the study (for example, achievement of milestones, percent of recruited patients and adverse events) as well as adherence to the study protocol. There will be two meetings between the investigator and the advisory board at enrollment start and at last recruitment. The committee itself has the right to actively address any study issues with the principle investigator.

On all relevant stages of the trial, safety aspects are considered and monitored by psychological and medical screening and ongoing monitoring with regard to adverse effects and adherence.

In general, we do not expect any severe adverse effects of the interventional approach since the intervention consists of well-established pain management strategies (IG) or information delivery (CG). So far, neither adverse side effects nor such negative experiences are reported in previous studies implementing the respective interventions [20–22, 43].

**Adverse events** (AEs), including chronic or recurrent physical and psychiatric illness, as well as all reports and results have to be documented in patient’s health records and case report forms. Documentation of AEs includes the type of AE, starting and ending point, severity, causality to intervention, intensity and result.

Adverse events may be reported by site investigator, study nurse and trainer during the course of the interventions and will be assessed in follow-up measurements from the first training session of the children. The ethics committee at the site, the principle investigator and the TSC will be informed immediately.

## Discussion

FAP represents a considerable strain on children and their families and causes increased healthcare utilization. In our previous study, children suffered for more than two years from FAP [19]. Current evidence suggests that psychological treatments can lead to a clinically significant reduction of pain and, almost certainly, of psychological strain too [13–18]. As increased health care utilization, especially the repeated diagnostic procedures, causes high health care costs ($6104.30 US per child; [5]) appropriate management of FAP is urgently needed and will reduce these costs substantially. Thus demonstrating efficacy and feasibility in a multicenter study, psychological treatments for FAP will be available for affected children and their families and could be easily implemented in clinical practice. Furthermore, psychological support is not part of the standard medical care for patients with FAP in Germany; therefore, participation in this multicenter study may improve the quality of patient care. Furthermore, the group setting offers an economically and emotionally advantageous approach.

### Generalizability

Participants are recruited from five pediatric gastroenterology outpatient clinics throughout Germany using consecutive and active recruitment strategies. This broad strategy and the specified sample characteristics with respect to age, gender and psychological strain allow recruiting of a representative sample of FAP patients. This will enhance external validity and enable generalization of the results.

### Dissemination

Beyond regular publication, the results of the trial will be used for dissemination of the intervention as well as for the establishment of international collaboration with related study groups to further disseminate the evidence-based treatment program and improvement of care for children with FAP. Collaborations with self-help organizations will enhance the dissemination of the results and the implementation of the treatment approach in clinical practice as well.

### Strengths of the trial

This trial will add to our current knowledge in several ways. First, the inclusion of an active control group instead of a waiting list control group is able to ensure that effects are due to treatment content, rather than to nonspecific patient-therapist factors [16]. Second, a 3- and 12-month follow-up will enhance our knowledge on the maintenance of treatment effects. Third, the implementation of the program in several centers will increase the generalizability of treatment effects. Fourth, including a broad range of psychosocial variables for children as well as their parents allows the detailed analysis of psychosocial treatment effects as well as the analysis of predictors and mediators.

## Trial status

The trial is currently recruiting.

## Electronic supplementary material

Additional file 1: **List of ethical bodies.** (DOC 15 KB)

## Abbreviations

AE: adverse events
CBT: cognitive-behavioral therapy
CG: control group
FAP: functional abdominal pain
IG: intervention group
ITT: intention to treat
PPA: per protocol analysis
TSC: trial steering committee
T1: point of measurement pre-intervention
T2: point of measurement postintervention
T3: point of measurement at 3-month follow-up
T4: Point of measurement at 4-month follow-up
