# Supplementary material for: Stop the pain: study protocol for a randomized-controlled trial
Source: Trials. 2014 Sep 11;15:357. doi: 10.1186/1745-6215-15-357 (PMC4171565; doi:10.1186/1745-6215-15-357)
Supplement: Supplementary file 1 — Additional file 1: List of ethical bodies. (DOC 15 KB) [file 13063_2014_2228_MOESM1_ESM.doc]

**Additional file 1: list of ethical bodies**

Ethical approval was given by the following institutions involved:

**Leading ethics comittee:**

Ethics committe of the University of Potsdam

Main vote: reference number:19/2013, date 30/05/201

Amendments: reference number 08/2014, date 02/04/2014 and final approval 13/05/2014

**Study Centres:**

Study centre: Princess Margaret Children’s Hospital Darmstadt

Ethical body: State Chamber of Physicians, Hessen

Main vote: reference number MC 69/2014, date 01/04/2014

Study centre: University Children’s Hospital Düsseldorf

Ethical body: Ethics Committee of the University of Düsseldorf

Main vote: study number 442R, registry ID: 2013091434, date: 27/01/2014

Study centre: Charité University Medicine, Department of Gastroenterology

Ethical body: Charité Ethics Committee

In this multicenter trial, the Charité Ethics Committee declares approval along with the votes by the leading ethics committee of the University of Potsdam as well as state chamber of physicians in Hamburg (date: 05/11/2013)

Study centre: University Medical Centre Ulm, Department of Paediatrics and Adolescent Medicine

Ethical body: Ethics Committee of the University of Ulm

Reference number 273/13, main vote date: 09/09/2013

Study centre: Catholic Children’s Hospital Wilhelmstift, Hamburg

Ethical body: State Chamber of Physicians, Hamburg

Main vote, date: 02/08/2013

Reference Number MC-214/13
